# Plant immune inducer ZNC promotes rutin accumulation and enhances resistance to *Botrytis cinerea* in tomato

**DOI:** 10.1007/s44154-023-00106-0

**Published:** 2023-08-22

**Authors:** Haipeng Zhao, Xiangyu Ding, Xiaomeng Chu, Haimiao Zhang, Xinyu Wang, Xinwen Zhang, Haoqi Liu, Xiaoying Zhang, Ziyi Yin, Yang Li, Xinhua Ding

**Affiliations:** 1 State Key Laboratory of Crop Biology, Shandong Provincial Key Laboratory for Biology of Vegetable Diseases and Insect Pests, College of Plant Protection, Shandong Agricultural University, Taian, 271018 Shandong P. R. China; 2 Shandong Pengbo Biotechnology Co., Ltd., Taian, 271000 China

**Keywords:** Endophytic fungus extract, Metabolome, Flavonoids, Tomato gray mold, JA Signaling, ROS

## Abstract

Gray mold is a destructive disease caused by *Botrytis cinerea*, a pervasive plant pathogen, which poses a threat to both tomato growth and postharvest storage. The utilization of induced resistance presents a potential strategy for combating plant pathogenic attacks. ZNC (zhinengcong), an extract derived from the endophytic fungus *Paecilomyces variotii*, has been discovered to play a vital role in preventing diverse forms of bacterial infections. Nevertheless, the precise mechanism behind its ability to enhance tomato resistance to fungi remains unclear. In this study, we found that the exogenous spraying of ZNC could significantly improve the resistance of tomato plants to *B. cinerea*. The results of both the metabolomic analysis and high-performance liquid chromatography (HPLC) demonstrated that tomato plants responded to ZNC treatment by accumulating high levels of rutin. Additional transcriptome analysis uncovered that rutin enhances tomato resistance possible by initiating the generation of reactive oxygen species (ROS) and phosphorylation of mitogen-activated protein kinases (MPKs) related genes expression during the initial phase of invasion by *B. cinerea*. In addition, we also found that rutin might activate plant immunity by eliciting ethylene (ET) and jasmonic acid (JA)-mediated pathways. Therefore, plant immune inducer ZNC and rutin has bright application prospects and high utilization value to control gray mold.

**Graphical Abstract:**

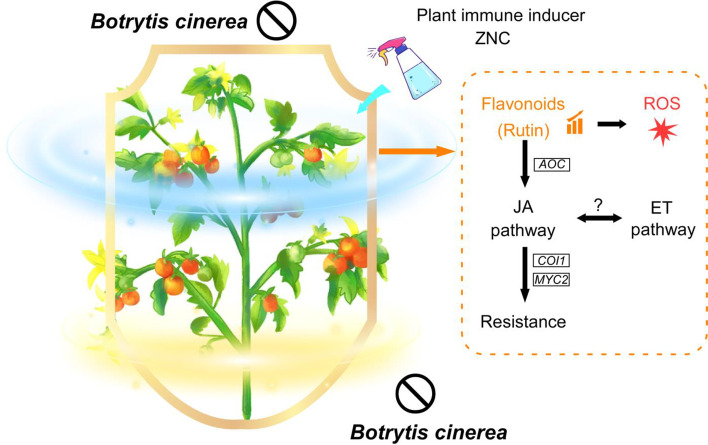

**Supplementary Information:**

The online version contains supplementary material available at 10.1007/s44154-023-00106-0.

## Introduction

Tomato, one of the most important vegetables around the world, contains abundant nutrients and secondary metabolites that are essential for the maintenance of human health (Testone et al. 2019; Vats et al. 2022). However, with the increase in plant production intensification rates and the gradual expansion of planting areas, tomatoes face an increasing variety of biotic stresses. Among these diseases, gray mold, which is caused by *B*. *cinerea*, stands out as one of the most detrimental afflictions affecting tomatoes, leading to significant economic losses both before and after harvest (Li et al. 2020; Soltis et al. 2019). Currently, the management of gray mold primarily relies on the use of chemical fungicides (Adnan et al. 2018). However, excessive or improper use of fungicides has exerted various deleterious responses, including evolved resistance to pathogens and environmental pollution (Mosbach et al. 2017). Therefore, new strategies for research and development of eco-friendly and efficient biological fungicides are needed to control this destructive pathogen.

Through the co-evolution with pathogens over millions of years, plants have evolved rigorous innate defense systems, including inducible defenses, to avoid disease infection (Ramirez-Prado et al. 2018). Inducible resistance pertains to the physiological and biochemical modifications observed in plants when exposed to stimuli from pathogenic organisms. These alterations enable plants to combat invading pathogens by inhibiting cell lesions, producing toxic metabolites, or inducing changes in both the quantity and quality of tissue components (Hammerschmidt et al., 1999; Parker et a., 2022). Recently, the manipulation of inducible plant defenses has received increased attention due to its importance for plant disease management. Exogenously applying synthetic plant defense elicitors, such as oligosaccharides, small-molecule metabolite elicitors, peptides, lipids, or their derivatives, has been demonstrated to significantly improve induced resistance in plants (Návarová et al. 2012). Examples of this include BcGs1 (a protein-like elicitor), lipopolysaccharide as a lipid elicitor, and β-glucan, which can effectively safeguard plants from pathogen infections by activating plant innate immunity (Ma et al. 2015; Ranf et al. 2015; Yamaguchi et al. 2000).

Changes in the growth and development of plants, as well as alterations in their environment, can result in changes in gene transcriptional expression (De Geyter et al. 2012). These changes ultimately lead to changes in metabolites, and the metabolome generally reflects the current state of the organism (Zhan et al. 2022). Primary metabolites are essential for plant normal growth, while secondary metabolites are more involved in responses to external stimuli, such as diseases (Erb and Kliebenstein 2020; Mellidou et al. 2021; Zhan et al. 2020). Plants have evolved multiple secondary metabolic pathways that produce a variety of novel substances to fight against invading pathogens. Among them, rutin, as a secondary metabolite of flavonoids, can scavenge free radicals and increase antioxidant levels (Qu et al. 2019). Recent research has demonstrated the crucial function of rutin in providing defense against various bacterial diseases, such as *Ralstonia solanacearum*, *Xanthomonas oryzae* pv. *Oryzae* (Xoo), *Xanthomonas oryzae* pv. *Oryzicola* (Xoc), and *Pseudomonas syringae* pv. tomato DC3000 (Lim and Li 2017; Yang et al. 2016). However, the effect of rutin on necrotrophic fungal plant pathogens such as *B. cinerea* has been little studied. Plants deploy diverse defense-signaling pathways to respond to biotic stresses. Emerging research suggests that the synthesis of secondary metabolites in plants is regulated by hormone-mediated signaling pathways. These immune pathways facilitate the transmission of stimulus signals via MAPK-dependent phosphorylation and other processes, leading to transcriptional reprogramming and the dynamic modulation of the immune system. This modulation is achieved through the regulation of salicylic acid (SA), ethylene (ET), jasmonic acid (JA), and reactive oxygen species (ROS) (Dong et al., 1998; Yuan et al. 2021; Zhou and Zhang 2020). Different pathogens usually induce hormonal crosstalk. In general, SA is often sensitive to biotrophic pathogens, whereas JA is usually related to plant defense against necrotrophic pathogens (Caarls et al. 2015; Meng et al. 2020).

ROS are a set of unstable molecules, including hydrogen peroxide (H_2_O_2_) and superoxide (O^2−^), that perform crucial roles in resistance against pathogens and cellular signaling (Mehdy et a., 1994). The generation of ROS occurs in plants as an early response to external stimuli. In most cases, phytopathogen infection results in a burst of ROS that leads to localized cell death (LCD) and prevents persistent pathogen infection (Nie et al. 2021; Wang et al. 2020). However, ROS are also broadly recognized as reactive particles harmful to cells as they damage intracellular proteins, lipids, and nucleic acids, and even can exacerbate the disease (Marschall and Tudzynski 2016; Rossi et al. 2011). For instance, in *Arabidopsis thaliana*, the ROS-mediated plant cell death did not prevent the infestation with the necrotrophic pathogen *B. cinerea* but promoted its colonization (Govrin and Levine 2000). Hence, effective antioxidant defense systems, such as scavenging enzymatic systems, including catalase (CAT) and ascorbate peroxidase (APX), play a crucial role in plants by preventing the production of ROS and consequently thwarting necrotrophic pathogens (Finiti et al. 2014; Unger et al. 2005).

ZNC is the fermentation product of the endophytic fungus *Paecilomyces variotii* (Lu et al. 2019). As an effective plant immunity elicitor, ZNC, at ultra-low concentrations, showed high activities by not only promoting plant root growth but also enhancing resistance to pathogenic bacteria as a result of the activation of ROS burst, callose deposition, and the upregulation of pathogenesis-related (PR) genes (Cao et al. 2021; Lu et al. 2019). However, the role of ZNC in combating necrotrophic fungal diseases remains unknown. In this study, the tomato–ZNC–*B. cinerea* interaction was used to develop a model experimental system. Using an integrated approach, the effect of the ZNC application on the enhancement of tomato resistance to *B. cinerea* and its underlying potential mechanism were comprehensively investigated. Our findings indicate that the application of exogenous ZNC through spraying significantly enhances plant resistance against *B. cinerea*. Metabolomic analyses revealed that tomato leaves treated with ZNC displayed an increased accumulation of diverse flavonoids, carbohydrates, amino acids, and their derivatives. Notably, the flavonoid rutin exhibited the highest level of accumulation. HPLC analyses corroborated these findings from metabolomic studies, demonstrating a substantial increase in rutin content following ZNC application. Further transcriptome analysis showed that rutin application triggered a burst of ROS, which was consistent with the results of rutin application in vitro. In addition, transcriptome and qRT-PCR analyses revealed that rutin might activate plant immunity by eliciting ethylene (ET) and jasmonic acid (JA)-mediated pathways. All these results indicated that the ZNC application inhibits tomato gray mold, and rutin, an immune-driven resistance agent, plays a dominant role in the ZNC-mediated *B. cinerea* control.

## Results

### ZNC can improve tomato resistance to *B. cinerea*

**Fig. 1:**
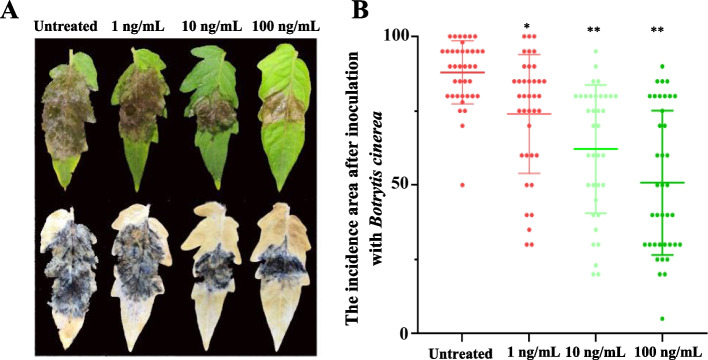
ZNC treatment was found to enhance the resistance of tomatoes against *B*. *cinerea*. **A** Tomato leaves were inoculated with *B*. *cinerea* using varying concentrations of ZNC. **B** The disease index of *B*. *cinerea* was measured and statistically analyzed for different ZNC concentrations. Each experiment was repeated more than 20 times, and the results are reported as the mean value ± standard deviation (SD). One-way ANOVA and Tukey post-hoc test for multiple comparisons were used to calculate significance, ** *p* < 0.01

Two-week-old tomato plants were treated with different concentrations of ZNC (1, 10, and 100 ng/mL) at weekly intervals. After four applications, the plants were inoculated with *B*. *cinerea*. The phenotypes were counted, and disease indices were calculated after 5 d of inoculation. The disease index of untreated plants was 98.68, whereas the disease indices of ZNC-treated plants at 1, 10, and 100 ng/mL were 88.82, 79.17, and 67.76, respectively. Compared to the control, tomato plants treated with different concentrations of ZNC showed significantly lower disease incidence and better resistance, especially with 10 ng/mL or 100 ng/mL of ZNC (Fig. 1A, B). In conclusion, in vitro spraying of ZNC could significantly improve tomato resistance to *B. cinerea*. For the sake of conserving resources and minimizing costs, we chose an experimental concentration of 10 ng/mL.

### ZNC promotes the accumulation of rutin

ZNC functions as a positive regulator of tomato resistance to *B cinerea*. We conducted metabolomics analysis on tomato leaves treated with 10 ng/mL ZNC after 24 h, using 6 biological replicates. Principal component analysis (PCA) was conducted on both cation and anion modes to aggregate the data from the same treatment. The results demonstrated clear separation between the control group and the ZNC-treated groups (Fig. S1A, B), indicating distinct accumulation patterns of metabolites in plants with and without ZNC treatment.

**Fig. 2:**
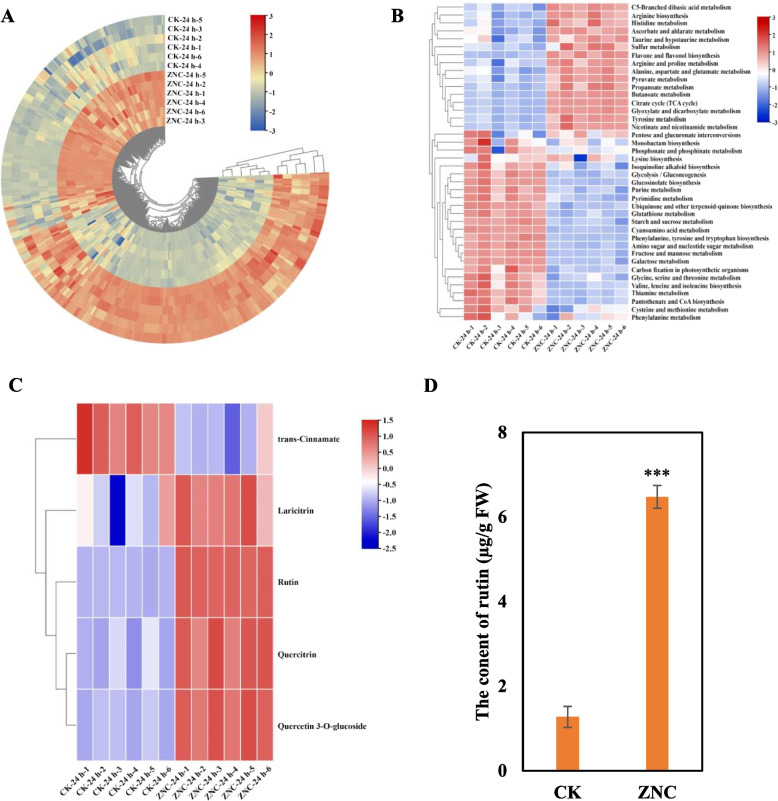
ZNC promotes the accumulation of flavonoids, especially rutin. **A** Enrichment of differential metabolites in plants treated with 10 ng/mL ZNC. **B** Enrichment of differential metabolic pathways in plants treated with 10 ng/mL ZNC. **C** Substances that vary in the flavone and flavonol biosynthesis pathways. **D** Determination of rutin content in tomato leaves after ZNC treatment. One-way ANOVA and Tukey post-hoc test for multiple comparisons were used to calculate significance, ** *p* < 0.01

The MetDNA (http://metdna.zhulab.cn/) database was used to match the experimental mass spectrometry data for the identification of metabolites, and the differentially expressed metabolites between the treatment and control group were analyzed. We identified a total of 351 annotated differential metabolite components, including amino acids, carbohydrates, organic acids in the primary metabolite pathway and phenols in secondary metabolites (Fig. 2A). These differential metabolites are enriched in 40 differential KEGG metabolic pathways, including but not limited to amino acid metabolism, glycolysis pathway, TCA cycle, biosynthesis of panquinone and other terpene quinones, ascorbic acid and aldehyde acid metabolism, phenylalanine metabolism, and flavonol biosynthesis pathways (Fig. 2B).

The metabolism of flavonoids and flavonols not only enhances plant resistance to stress but also serves as a defense mechanism against plant pathogens. This is achieved through the induction of jasmonic acid (JA) biosynthesis, transcriptional upregulation of defense response genes, and the synthesis of defense-related proteins (Dong et al. 2020; Liu et al. 2020). In this pathway, the synthesis of rutin, quercetin, quercetin-3-O-glucoside, and laricitrin was significantly induced, with the most pronounced changes in rutin (Fig. 2C). The content of rutin in tomato leaves treated with 10 ng/ml ZNC after 24 h was determined by HPLC, and the content of rutin in the leaves treated with ZNC showed a 5.08-fold increase than the control group (Fig. 2D). Therefore, ZNC promoted the biosynthesis and accumulation of rutin.

### Rutin improves resistance to *B. cinerea* in tomato leaves and fruits

**Fig. 3:**
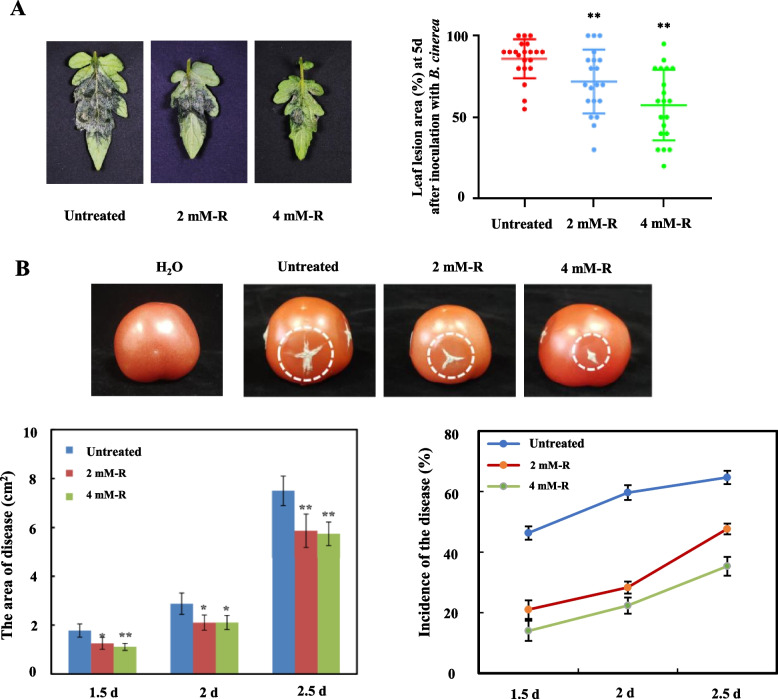
Different concentrations of rutin could improve the resistance of tomato leaves and fruits against *B*. *cinerea*. **A** Impact of different rutin concentrations on tomato leaves*.* **B** Effect of different rutin concentrations on tomato fruits*.* Each experiment was repeated more than 20 times, and the results are presented as the mean value ± standard deviation (SD). Significance was assessed using one-way ANOVA and Tukey post-hoc test for multiple comparisons, with * indicating *p* < 0.05 and ** indicating *p* < 0.01

We performed a plate-based antimicrobial assay and observed no significant difference in the diameter of the mycelial colonies between the treatment group supplemented with rutin and the control group. This finding suggests that rutin does not exert an inhibitory effect on the growth of *B. cinerea* (Fig. S2A, B). Tomato leaves were uniformly sprayed with the 2 mM and 4 mM rutin solutions for 2 h, then inoculated with *B. cinerea*, 5 d (after inoculation) later, the affected areas were measured. These results suggested the application of 2 mM and 4 mM rutin solutions through spraying resulted in significantly smaller lesion areas on tomato leaves compared to untreated plants (Fig. 3A). The lesion area of fruit sprayed with rutin was smaller than the control group, significantly (Fig. 3B). Moreover, rutin enhanced the resistance of tomato fruit to *B. cinerea*. It is worth noting that rutin does not act as a fungicide by directly inhibiting the growth of the pathogen. Instead, it functions as a plant immunity inducer, reducing the occurrence of the disease by inducing resistance against *B. cinerea* in both leaves and fruit.

### Rutin enhances *B. cinerea* resistance by regulating the ROS accumulation in tomato

**Fig. 4:**
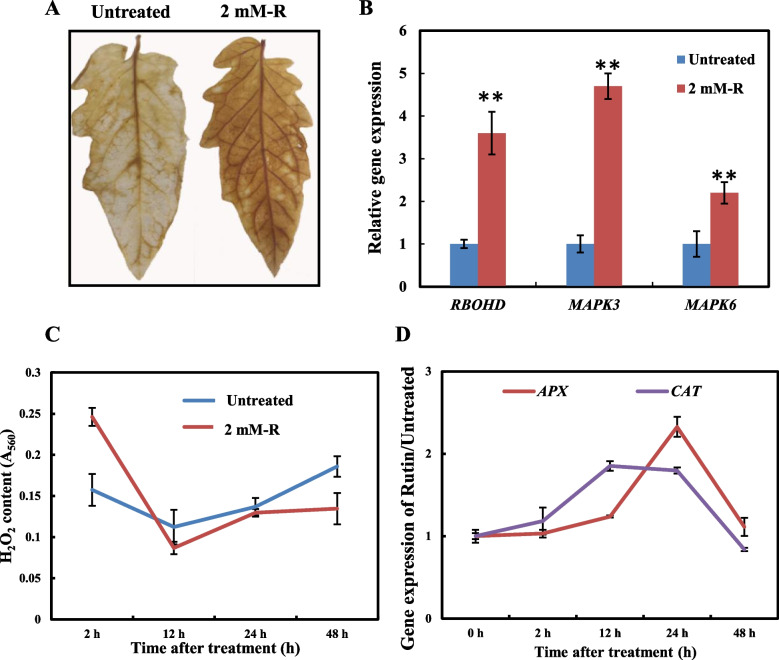
Immune response of tomato against *B*. *cinerea* infection*.* **A** DAB staining of leaves after 2 h rutin treatment. **B** Alterations in the expression of immune-related genes in tomato following 2 h rutin treatment. **C** Accumulation of hydrogen peroxide in *B*. *cinerea*-infected tomato. **D** Changes in the expression of antioxidant enzyme genes in *B*. *cinerea*-infected tomato*.* Each experiment was replicated three times, and the results are presented as the mean value ± standard deviation (SD). Significance was determined using one-way ANOVA and Tukey post-hoc test for multiple comparisons, with * indicating *p* < 0.05 and ** indicating *p* < 0.01

To elucidate the *B. cinerea* resistance mechanism induced by rutin in tomatoes, ROS generation in tomato leaves was detected after spraying rutin for 2 h using DAB staining. The results of the DAB staining method showed a significant accumulation of H_2_O_2_ in leaves (Fig. 4A), and the early immune-related genes (*RBOHD*, *MAPK3*, and *MAPK6*) were significantly upregulated to a higher expression level (Fig. 4B). These results showed, rutin enhanced the *B. cinerea* resistance by inducing an early innate immune response. After the treatment of tomato leaves with rutin for 12 h, the plants exhibited significantly reduced H_2_O_2_ contents compared to the control until 48 h of treatment (Fig. 4C). The expression levels of antioxidant-related genes (*APX* and *CAT*) in rutin-treated leaves were significantly upregulated to a greater extent (Fig. 4D). Therefore, rutin improved the *B. cinerea* resistance in tomatoes, probably by regulating the content of hydrogen peroxide.

### Ethylene (ET) signaling is involved in rutin-mediated *B. cinerea* resistance

**Fig. 5:**
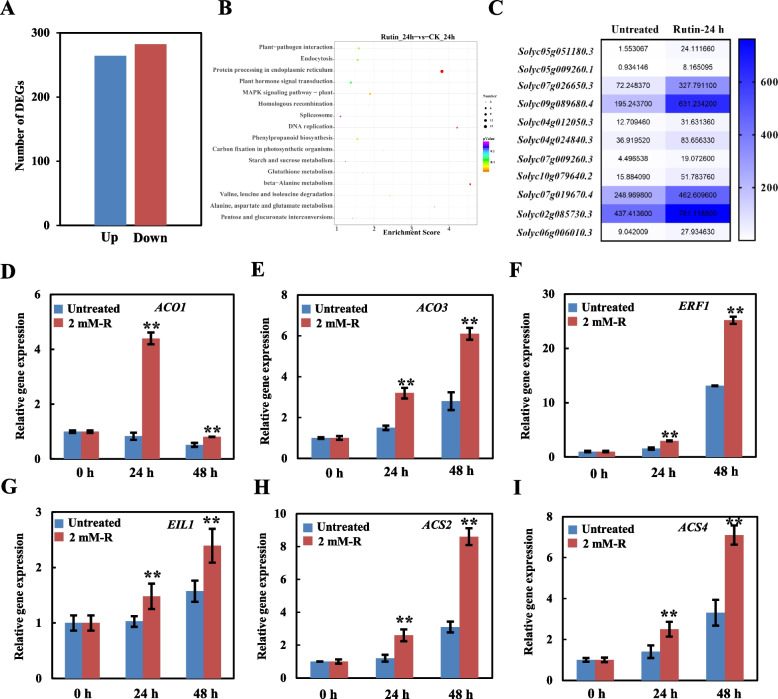
A summary of rutin-induced changes in gene expression in tomato leaves. **A** Differential gene expression at 24 h after rutin treatment. **B** Heatmap representation of hormone-related gene expression at 24 h after rutin treatment. **C** Enrichment analysis of metabolic pathways affected by rutin treatment.** D**, **E**, **F**, **G**, **H**, and **I** alterations in the expression of genes related to the ethylene (ET) pathway. Each experiment was performed three times, and the results are presented as the mean value ± standard deviation (SD). Significance was determined using one-way ANOVA and Tukey post-hoc test for multiple comparisons, with ** indicating *p* < 0.01

In order to examine the role of rutin in regulating gene expression at the transcriptional level, we performed transcriptome analysis on tomato leaves treated with a concentration of 2 mM rutin. The analysis was conducted at two time points: 0 h and 24 h after treatment, using RNA sequencing. Following 24 h of rutin treatment, we observed that 264 genes were upregulated, while 282 genes were downregulated (Fig. 5A). The KEGG enrichment analysis demonstrated that rutin primarily influenced pathways such as flavonoid biosynthesis, plant-pathogen interaction, and MAPK signaling (Fig. 5B). Furthermore, genes such as *ERF1B*, *ABR1*, *ACO1*, and *ILL6* (Fig. 5C), which are known to regulate disease resistance, exhibited a significant upregulation in response to rutin treatment. These findings suggest that rutin modulates the expression levels of genes involved in disease resistance, potentially through hormone synthesis pathways.

Studies have indicated that the ethylene (ET) signaling pathway plays a crucial role in the innate immune system, activating defense responses against necrotrophic organisms. ET positively regulates the expression of genes involved in JA synthesis, such as allene oxide synthase (AOS). Additionally, MeJA (methyl jasmonate) can induce the expression of *ACC* (1-aminocyclopropane-1 carboxylic acid) and *ACO* (aminocyclopropane-1 carboxylic oxidase), thereby promoting ethylene production. Considering that some ET-related genes (*ACO1* and *ERF1B*) showed significant induction under the rutin treatment, we screened the expression level *ACO1*, *ACO3*, *ERF1*, *EIL1*, *ACS2*, and *ACS4*, which was related to ET signaling pathway, and all these genes were similarly upregulated, indicating that ET was involved in the rutin-mediated resistance to *B*. *cinerea* (Fig. 5C-I).

### Rutin enhances tomato *B. cinerea* resistance by activating JA signaling

**Fig. 6:**
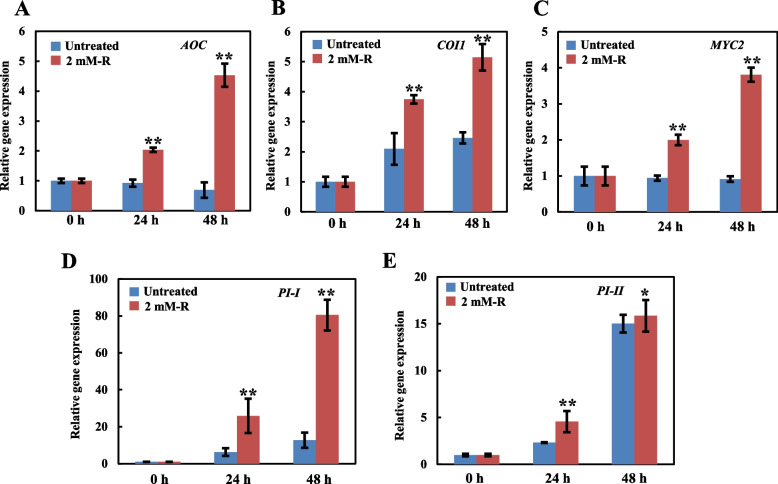
Expression levels of genes related to the jasmonic acid (JA) pathway in tomato leaves treated with rutin. **A**, **B, C**, **D**, and **E** represent the changes in the expression of JA pathway-related genes. Each experiment was performed three times, and the results are presented as the mean value ± standard deviation (SD). Significance was determined using one-way ANOVA and Tukey post-hoc test for multiple comparisons, with ** indicating *p* < 0.01

Crosstalk between plant hormones play an important role in the resistance against plant diseases. Moreover, JA and ET signaling pathways are usually connected to the resistance to necrotrophic pathogens (Jones and Dangl 2006; Wu et al. 2022). Synergistic effects of JA and ET against these pathogens exist. In *Arabidopsis*, the synergistic effect of ET and MeJA enhanced its *B. cinerea* resistance. To analyze whether the rutin-mediated resistance to *B. cinerea* in tomato plants is also related to the JA signaling pathway, changes in the JA-related genes expression level were examined. The results showed that rutin did not induce changes in the expression of hormone-related genes in tomato plants that were not inoculated, as compared to the control group. However, *B. cinerea* induced the upregulated expression of JA-related genes significantly (Fig. 6) at 48 h after inoculation, indicating a direct relationship between the rutin-mediated *B. cinerea* resistance and JA signaling.

**Fig. 7:**
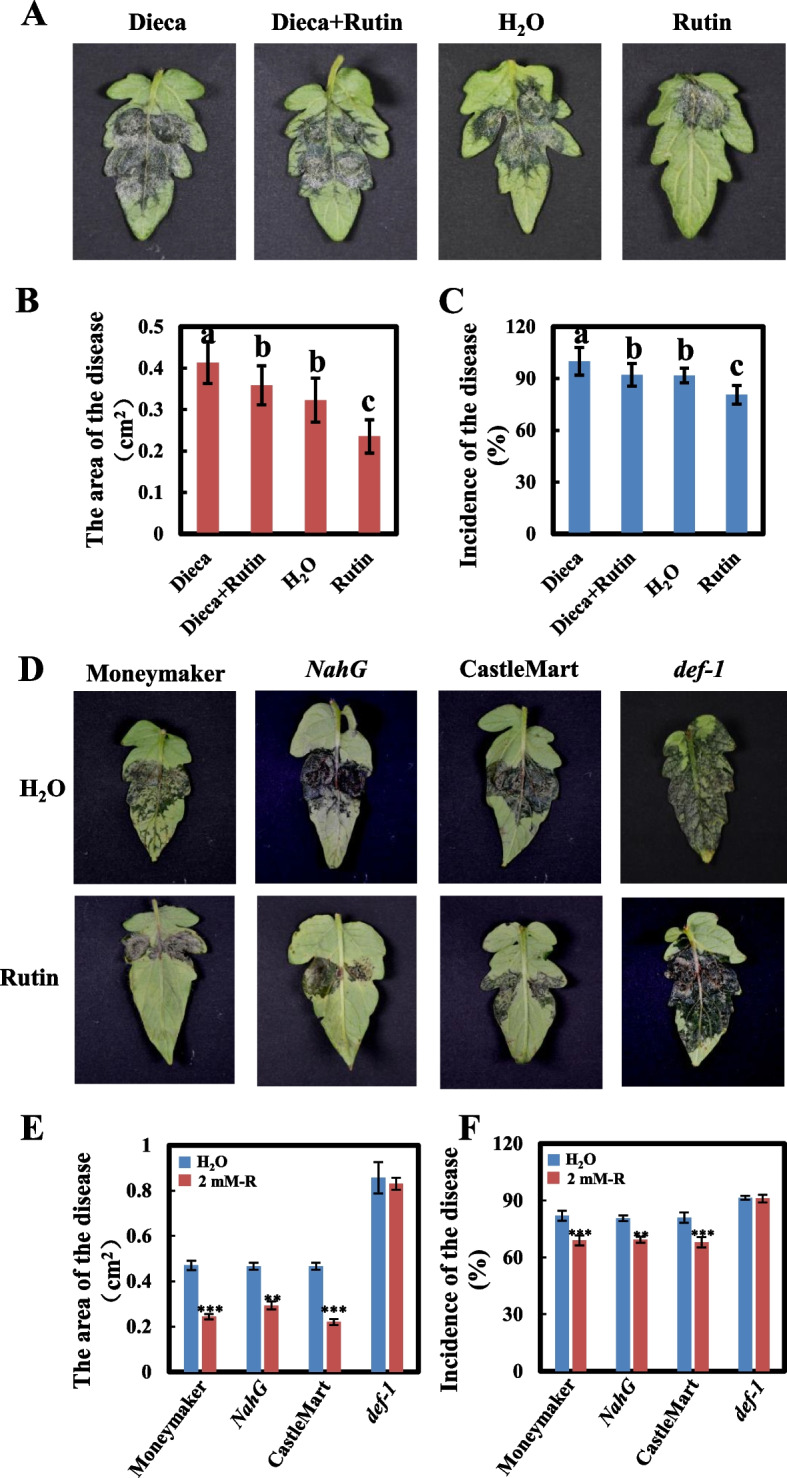
Impact of Dieca treatment on tomato resistance against *B*. *cinerea*. **A** Visual representation of *B*. *cinerea* inoculation. **B** Quantification of disease area following *B*. *cinerea* inoculation. **C** Incidence statistics of *B*. *cinerea* inoculation

To confirm that the *B. cinerea* resistance mediated by rutin is related to JA signaling, tomato plants were treated with Dieca, a JA synthesis inhibitor, for 24 h and then inoculated by *B. cinerea*. 5 d of inoculation later, the lesion area was measured, and the incidence rates were calculated. The lesion areas of plants treated with Dieca, Dieca + Rutin, H_2_O, and 2 mM rutin were 0.41, 0.36, 0.32, and 0.23 cm2, respectively (Fig. 7A, B), and their incidence rates were 100%, 92.11%, 91.67%, and 80.56%, respectively (Fig. 7A, C). The results suggested that, the lesion areas and the incidence of disease in inoculated plants significantly increased after the Dieca treatment than the control treatment, indicating that JA plays an important role in *B. cinerea* resistance of tomato plants. There was nearly no difference between the Dieca + Rutin treated plants, and the H_2_O-treated plants (Fig. 7A, B, and C), indicating that the inhibition of JA synthesis could abrogate tomato *B. cinerea* resistance. Furthermore, we conducted an analysis of disease severity in the *def-1* mutant (which exhibits a deficiency in JA accumulation) and its corresponding wild-type CastleMart, using the *NahG* plant (a transgenic Moneymaker plant line that lacks SA accumulation) as a negative control. As depicted in Fig. 7D, 48 h after inoculation, both the wild-type plant strains (Moneymaker and CastleMart) and the *NahG* plant exhibited smaller disease lesions when treated with rutin compared to the untreated control. In contrast, the *def-1* mutant displayed no significant difference in lesion size with or without rutin treatment (Fig. 7D, E, F). These findings indicate that the *B. cinerea* resistance mediated by rutin is dependent on the JA signaling pathway.

## Discussion

**Fig. 8:**
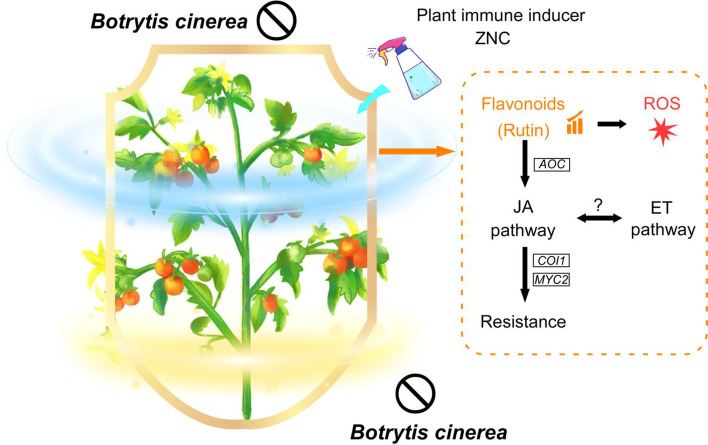
ZNC treatment enhances the accumulation of flavonoids and increases tomato resistance against *B*. *cinerea*

Plants face a variety of pathogens during their growth and development and have evolved sophisticated defense systems to cope with these stresses, including induced defenses (Jones and Dangl 2006). Our previous studies have shown that ZNC is a highly effective immunostimulant that can protect plants from bacterial diseases (Cao et al. 2021; Peng et al. 2020). Gray mold caused by the fungal pathogen *B. cinerea*, is a worldwide destructive disease in tomato production and quality (Li et al. 2020; Soltis et al. 2019). In the present study, we found that ZNC can also increase *B. cinerea* resistance in tomato. Integration of metabolomic analysis with HPLC results confirmed the enrichment of rutin in tomato leaves following ZNC treatment. Furthermore, it was demonstrated that rutin exhibited a potent induced resistance effect against *B. cinerea* in both fruits and leaves. Considering the transcriptional data, our findings suggest that rutin possesses the ability to stimulate the plant defense response in tomatoes. This response is closely associated with the burst of ROS and the expression of genes related to the ET and JA signaling pathways. These findings provide initial insights into the mechanism underlying ZNC-induced resistance in tomatoes against gray mold (Fig. 8).

Notably, ZNC can promote plant nutrient uptake and auxin biosynthesis after repeated application. In a short period of time after application, it is able to affect hormonal changes and signaling pathways in *Arabidopsis* (Lu et al. 2019). Our experimental data also indicated that 1 and 10 ng/mL ZNC could promote tomato growth parameters, including height, stem diameter, root length and root weight (data not shown). However, they may work in different ways to improve tomato resistance to *B. cinerea*. We speculate that the former resists pathogens by enhancing the plant's own root system and growth vigor. The latter is to resist pathogen invasion by rapidly activating disease-resistant signals and synthesizing resistance-related metabolites. However, the specific mechanism of action difference between the two still needs further verification.

Rutin is a diverse group of flavonoids that are commonly found in everyday plant-based foods, including fruits and vegetables (Chua 2013). Rutin serves not only as a nutrient but also possesses anti-inflammatory properties and is recognized for its high antioxidant capacity. Moreover, it can be employed as an activator to enhance plant disease resistance (Bika et al. 2021; González-Domínguez et al. 2019; Jantrawut et al. 2017). Rutin can promote the growth and biomass of *Amaranthus hypochondriacus* K472 and improve the absorption capacity of K472 for cadmium (Yang et al. 2022). External spraying of rutin can also inhibit the proliferation of *Xanthomonas oryzae pv. oryzae* and improve the resistance of rice to it (Yang et al. 2016). While previous studies have shown that the external application of rutin enhances plant resistance to bacteria by upregulating the expression of genes associated with SA and the reactive oxygen species signaling pathway (Yang et al. 2016), the precise mechanism by which rutin augments the antifungal effect in plants remains largely unexplored. Here, we found that rutin can also increase tomato resistance to fungus *B. cinerea* by stimulating reactive oxygen species and activating plant ET and JA-dependent signaling pathways.

Plants possess the ability to detect pathogen-associated molecular patterns (PAMPs) or effector molecules, triggering pattern-triggered immunity (PTI) and effector-triggered immunity (ETI) (Zhang et al. 2020). These defense mechanisms share common immune responses, including the activation of disease-related proteins (PRs), generation of reactive oxygen species (ROS) (Marschall and Tudzynski 2016), deposition of callus (Li et al. 2019), and accumulation of secondary metabolites (Su et al. 2021). ROS plays a crucial role as a defense signal in response to pathogen attacks, triggering allergic reactions and programmed cell death to hinder pathogen spread. However, in the case of necrotrophic fungi, pathogens can exploit the accumulation of ROS to achieve full pathogenicity (Yu et al. 1998). In this study, we found that rutin treatment significantly enhanced the expression of antioxidant-related genes, such as *APX* and *CAT*, while reducing H_2_O_2_ levels. These findings contribute to the enhanced resistance of tomato plants to *B. cinerea*. Nonetheless, further research is required to elucidate the mechanisms underlying ROS production and scavenging during different stages of plant defense and their coordination with other signaling pathways.

Studies are increasingly showing that the application of exogenous biological control agents enhances plant resistance to disease, mainly through increased accumulation of compounds. Exogenous spraying with strigolactone (SL) increased the activity of peroxidase and catalase and improved the ability of apple seedlings to withstand drought stress (Zheng et al. 2021). The treatment of cotton seedlings with 24-epibrassinolide (EBR) improved the cold resistance of cotton seedlings by inhibiting the signal transduction of ABA and ETH (Dou et al. 2021). In this study, metabolome analysis showed that the accumulation of metabolites was quite different under the conditions with and without ZNC. HPLC assay confirmed that the rutin content in the tomato leaves treated with ZNC was 5.08 times than control, suggesting that ZNC could induce the accumulation of immune substances (rutin) in plants, thereby increasing the resistance of plants to pathogens.

Plant defense against pathogens depends mainly on the crosstalk between the phytohormones SA, JA, and ET (Courbier et al. 2021; Peng et al. 2021; Song et al. 2022). Numerous studies have documented the involvement of the JA/ET signaling pathway in the elicitation of plant defense responses against *B. cinerea* (Shu et al. 2021; Vuorinen et al. 2021; Yang et al. 2020). Our study also showed that rutin treatment increased the expression level of genes in JA synthesis pathway, possibly by positively regulating the synthesis of JA, to achieve *B. cinerea* resistance in which the signaling pathway ET was also activated. In addition, leaves showed more sensitivity to the *B. cinerea* infection, when JA synthesis was inhibited, suggesting that this process is dependent on JA signaling. However, the relationship between JA and ET or other unknown regulatory pathways is still unknown.

To conclude, this study reveals that ZNC treatment leads to a significant increase in rutin content in tomatoes. Moreover, the external application of rutin through spraying stimulates the production of reactive oxygen species and enhances resistance against *B. cinerea* by upregulating the expression of genes related to ET and JA signaling pathways. Therefore, as an immune inducer, rutin may have an active function in the prevention and treatment of *B. cinerea*. However, the role of ROS production and scavenging mechanisms at different stages, the direct synergy between JA and ET, and coordination with other signals need to be further explored.

Rutin, a secondary metabolite flavonoid, plays a crucial role in enhancing the resistance of tomatoes against *B. cinerea*. It can act as an immune-inducer when applied before harvest to ensure the normal growth of tomatoes. Additionally, it can also inhibit the occurrence of *B. cinerea* after harvest, thus reducing the pathogenic process during transportation and storage of fruits and minimizing economic losses. Although the effectiveness of this ingredient is promising, further research is required to fully understand its processing and potential use. Additionally, it can be utilized in combination with other active ingredients to create sprays and solutions that enhance the resistance of tomatoes against *B. cinerea* in various ways.

## Materials and methods

### Plant material and growth conditions

Tomato plants (*Solanum lycopersicum* 'cv. Ailsa Craig’) were cultivated in a greenhouse under controlled conditions, with a temperature of 28℃ and 70% humidity. The plants were exposed to daily light/dark cycles of 16 h and 8 h, respectively. ZNC, an ethanol extract derived from *Paecilomyces variotii*, was obtained from Shandong Pengbo Biotechnology Company, located in Tai’an, Shandong Province, China. Rutin was procured from Aladdin Company in Shanghai, China. Rutin was dissolved in the 1 M NaOH solution to obtain a final concentration of 1 mol/L.

### Cultivation of* B. cinerea* and spore production

The colonies of *B. cinerea* were cultured on the PDA medium using a sterilized needle on an ultra-clean sterile workbench and then subcultured in an incubator at 25 °C. The mycelium reached the edge of the medium after about 5–7 days of growth, and the holes were evenly punched with a 0.5-mm puncher; thereafter, the clumps were picked with tweezers to inoculate the tomato leaves. Spores were produced after about 14 days, washed with 5 mL of sterilized ddH_2_O, filtered with nylon cloth, and counted using a hemocytometer under a microscope. The spore suspension was diluted to a final concentration of 1 × 10^5^ cfu/mL and inoculated onto tomato fruits.

### Pathogen inoculation method

After ZNC application, detached leaves were inoculated using the third and fourth leaves from the top. Plants and fallen leaves were preserved in a high humidity growth chamber at 25℃. The phenotype was observed and the disease index was calculated 48 h after inoculation. Six-week-old tomato leaves were uniformly sprayed with the 2 mM and 4 mM rutin solutions for 2 h then inoculated with *B. cinerea*, 5 d (after inoculation) later, the affected areas were measured. Similarly, the red ripe fruit was soaked in the rutin solution with final concentrations of 2 mM and 4 mM for 10 min. To inoculate the fruit, use a needle to create a small hole and inject 10 µL of 1 × 10^5^ cfu/mL spore suspension into the hole using a pipette. The lesion area was measured at 36 h,48 h and 60 h and take photos at 48 h.

### ROS detection

Two hours after rutin spraying, 3,3-diaminobenzidine (DAB) staining was used to detect the the accumulation of H_2_O_2_ in tomato leaves, and RNA was extracted to detect the expression of early immune-related genes (*RBOHD*, *MAPK3* and *MAPK6*). Tomato leaves were immersed in a solution of DAB (1 mg/mL), subjected to vacuum infiltration for 30 min, and then washed three times. The leaves were exposed to light illumination at 28 °C for 8 h to allow H_2_O_2_ to react. Chlorophyll was removed by immersing the leaves in boiling ethanol (95%) for 10 min. The concentration of H_2_O_2_ was determined using a hydrogen peroxide detection kit (Beyotime, Shanghai, China) following the manufacturer's instructions.

### Plant total RNA Extraction and quantitative real-time PCR

Total RNA was extracted from 100mg of fresh tissue per plant using the Plant RNA Kit R6827 (OMEGA Bio-Tek, USA), following the manufacturer’s protocol. Reverse transcription was performed using Evo M-MLV Reverse Transcriptase (Accurate Biology, China). In this study, approximately 5 ng of cDNA was employed as the template for qRT-PCR. The expression levels of various genes were normalized using the Eq. 2^–ΔΔCt^ method. qRT-PCR reactions were performed using the CFX96 Real-Time PCR detection system (Bio-Rad) with three technical replicates (Liu et al. 2022).

### Extraction of metabolite from tomato and detection

Metabolites were extracted from tomato leaves treated with 10 ng/mL ZNC after 24 h. 100 mg of fresh tomato leaves were ground using liquid nitrogen and then mixed with 1.0 mL of 70% methanol in water. The mixture was left to extract for 10 h at 4°C and then centrifuged at 12,000 rpm for 10 min at the same temperature. The supernatant was then filtered using an organic filter (0.22 μm) and transferred into an injection bottle. Around 10 µL of each sample was taken and mixed as pooled quality control (QC) samples. The samples were stored at –80 °C before LC–MS/MS analysis, for which, chromatographic separations were done with the ultra-performance liquid chromatography (UPLC) system (SCIEX, UK). Samples were injected into an ACQUITY UPLC T3 column (100 mm * 2.1 mm, 1.8 µm, Waters, UK) with a flow rate of 0.4 mL/min, and the column temperature was kept at 35 °C continuously. The mobile phase containing solvent A (0.1% formic acid in water) and solvent B (0.1% formic acid in acetonitrile). Gradient elution conditions were set as follows: 0 ~ 0.5 min 5% B; 0.5 ~ 7 min 5% to 100% B; 7 ~ 8 min 100% B; 8 ~ 8.1 min 100% to 5% B; 8.1 ~ 10 min 5% B. Each sample to be injected was 2 µL in volume. The mass spectrometer TripleTOF 5600plus (SCIEX, UK) was used to check metabolites eluted from the column, with both the positive and negative polarity modes with spray voltages of 5,000 and 4,500 kV, respectively. The curtain gas was presumed at 30 P.S.I., and the Ion source gas1 was presumed at 60 P.S.I., while the Ion source gas2 was set at 60 P.S.I., and an interface heater temperature was set at 650 °C. The LC–MS raw data files were converted into mzXML format and then deal with the XCMS software. Each ion was identified by combining the retention time (RT) and *m/z* data. The intensities of each peak were recorded, and the resulting three-dimensional matrix containing arbitrarily assigned peak indices (retention time-*m/z* pairs), sample names (observations), and ion intensities (variables) was generated. Total ions current chromatograms (TICs) and *m/z*-rt images of QC samples were exported to summarize the metabolite profiles of all samples and calculate the area of each chromatographic peak (Fig. S1). The online metDNA, KEGG and HMDB databases were used to annotate the peaks in the metabolites through matching the exact molecular masses (*m/z*) of each sample with the database. Metabolome data were log2-transformed for statistical analysis to improve normality and normalized. Hierarchical clustering and PCA were performed using R (www.r-project.org/) software with default settings. Metabolite identification and mapping performed using metDNA (http://metdna.zhulab.cn/) and MetaboAnalyst (MetaboAnalyst). For comparison of individual treatments with their relevant controls, unpaired two-tailed Student’s t-tests were used, and *P* ≤ 0.05 was considered significant (Shen et al. 2019).

Rutin was quantified by high-performance liquid chromatography (HPLC, Agilent Technologies 1200 series) with a column (Agilent Technologies ZORBAX SB-C18 4.6 × 250 mm). 0.1% acetic acid solution was used as the aqueous phase A, while the pure methanol was used for organic phase B. The ultraviolet (UV) chromatograms were recorded at 325 nm, and the column temperature was 35℃. A 1 mg/mL standard solution of rutin (Sigma, USA) was prepared with 100% chromatographic grade methanol and stored in a brown bottle at -20 °C.

### Statistical analysis

Tukey’s multiple range test or Student’s t-test was used for the statistical analysis by GraphPad Prism8 software. Statistical significance between the means of treatment groups was determined *p* < 0.05 (*) and *p* < 0.01 (**).

### Supplementary Information

**Additional file 1:** **Fig. S1.** (A) Principal component analysis (PCA) was conducted on the identified metabolites in positive mode using metID. (B) Principal component analysis (PCA) was performed on the identified metabolites in negative mode using metID.

**Additional file 2:** **Fig. S2.** Effects of different concentrations of rutin on the growth of B. cinerea. Each experiment was repeated three times (*n* = 3) and the results are presented as mean values ±standard deviation (SD). Significance was determined using one-way ANOVA followed by Tukey's post-hoc test for multiple comparisons.

**Additional file 3:** **Fig. S3.** Effects of different treatments in tomato.

**Additional file 4:** **Table S1.** Primers used in this study.

## Data Availability

The data that support the findings of this study and the materials used during the current study are available from the corresponding author on reasonable request.

## Abbreviations

*ACC*: 1-Aminocyclopropane-1- carboxylic acid
*ACO*: 1-Aminocyclopropane-1- carboxylic oxidase
AOS: Allene oxide synthase
APX: Ascorbate peroxidase
CAT: Catalase
DAB: 3,3-Diaminobenzidine
ET: Ethylene
HPLC: High-performance liquid chromatography
JA: Jasmonic acid
LCD: Localized cell death
MeJA: Methyl jasmonate
MPKs: Mitogen-activated protein kinases
PCA: Principal component analysis
PR: Pathogenesis-related
ROS: Reactive oxygen species
SA: Salicylic acid
TICs: Total ions current chromatograms
UPLC: Ultra-performance liquid chromatography
Xoo: *Xanthomonas oryzae* pv. *Oryzae*
Xoc: *Xanthomonas oryzae* pv. *Oryzicola*
ZNC: Zhinengcong
