# Supplementary figures and images for: Plant immune inducer ZNC promotes rutin accumulation and enhances resistance to Botrytis cinerea in tomato
Source: Stress Biol. 2023 Aug 22;3(1):36. doi: 10.1007/s44154-023-00106-0 (PMC10444710; doi:10.1007/s44154-023-00106-0)

A

Scores Plot

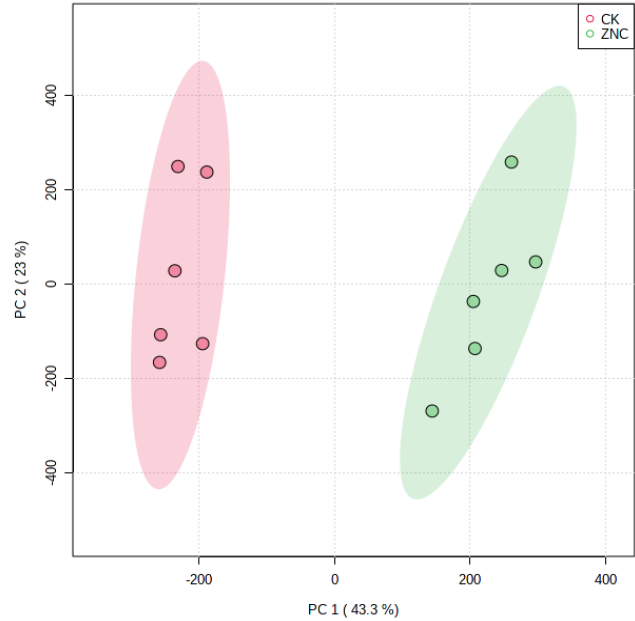

B

Scores Plot

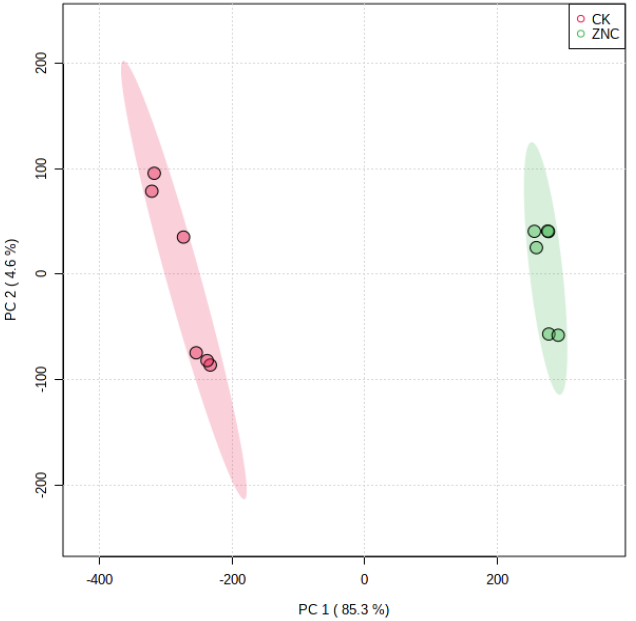

Supplement: Supplementary file 1 — Additional file 1: Fig. S1. (A) Principal component analysis (PCA) was conducted on the identified metabolites in positive mode using metID. (B) Principal component analysis (PCA) was performed on the identified metabolites in negative mode using metID. [file 44154_2023_106_MOESM1_ESM.pdf]

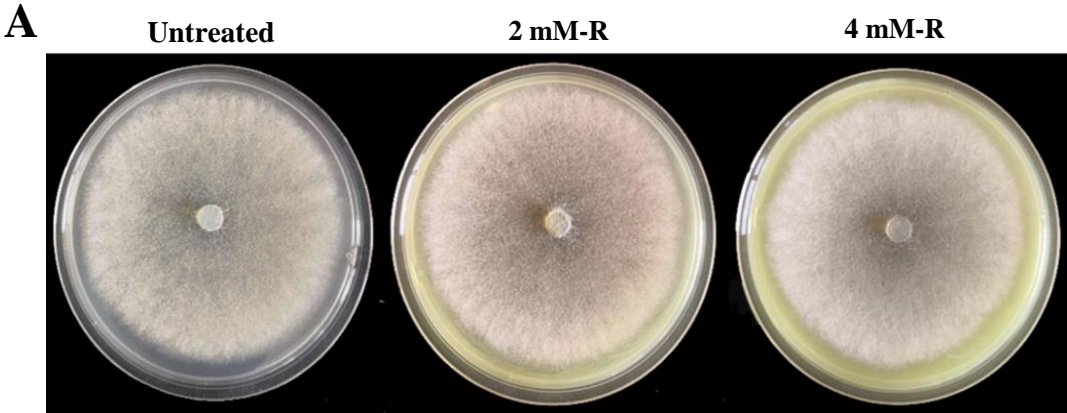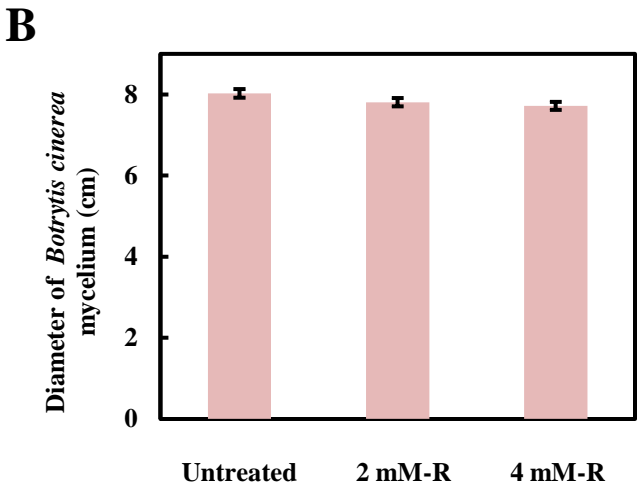

Supplement: Supplementary file 2 — Additional file 2: Fig. S2. Effects of different concentrations of rutin on the growth of B. cinerea. Each experiment was repeated three times (n = 3) and the results are presented as mean values ±standard deviation (SD). Significance was determined using one-way ANOVA followed by Tukey's post-hoc test for multiple comparisons. [file 44154_2023_106_MOESM2_ESM.pdf]

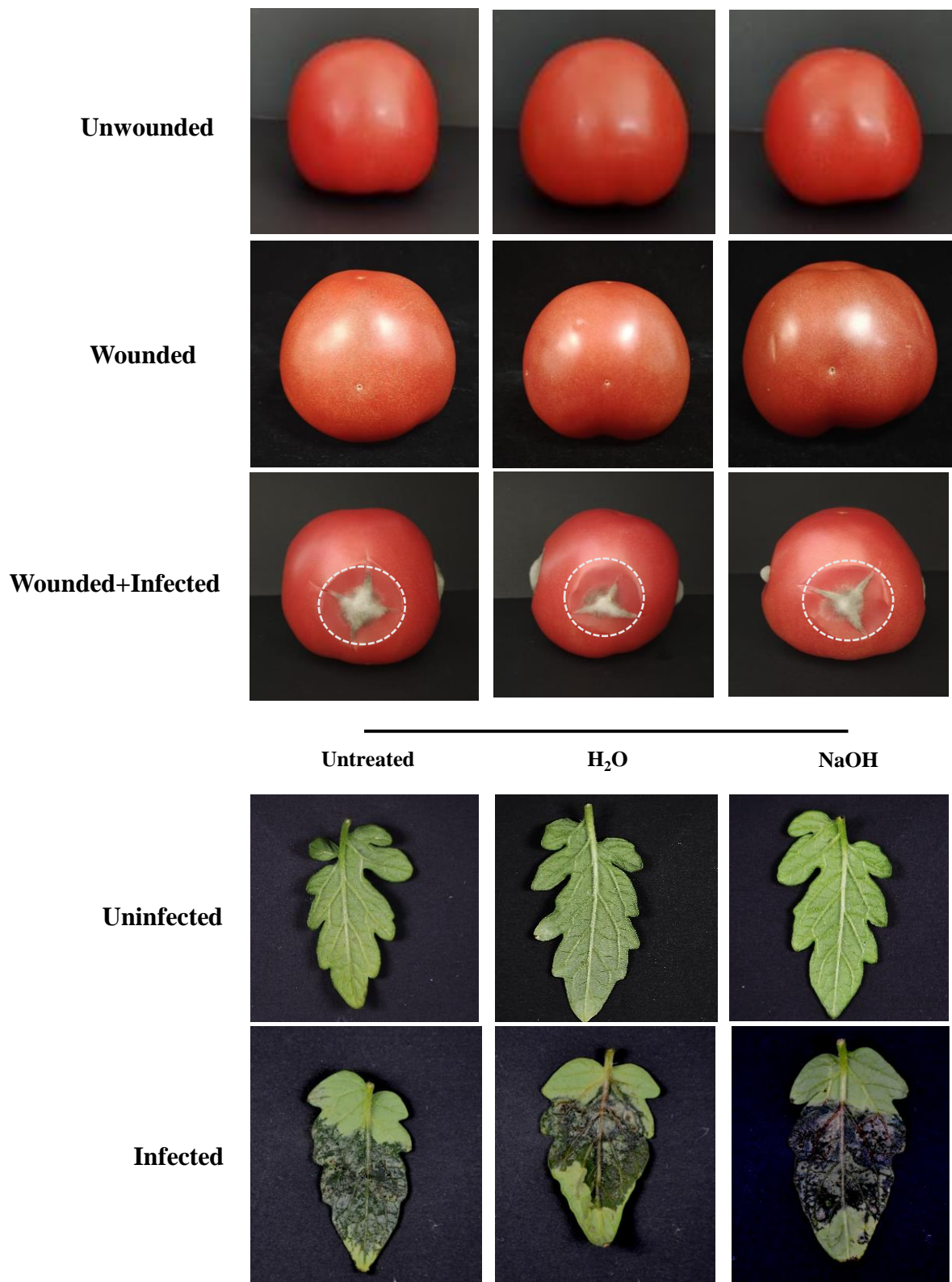

Supplement: Supplementary file 3 — Additional file 3: Fig. S3. Effects of different treatments in tomato. [file 44154_2023_106_MOESM3_ESM.pdf]
