# Supplementary material for: Plant immune inducer ZNC promotes rutin accumulation and enhances resistance to Botrytis cinerea in tomato
Source: Stress Biol. 2023 Aug 22;3(1):36. doi: 10.1007/s44154-023-00106-0 (PMC10444710; doi:10.1007/s44154-023-00106-0)
Supplement: Supplementary file 4 — Additional file 4: Table S1. Primers used in this study. [file 44154_2023_106_MOESM4_ESM.pdf]

Table S1. qRT-PCR Primers

Gene	Primers	Sequence
RBOH1	RT-F	GTTGCTGCAGCCATTGTCAC
	RT-R	GGCTTGGGCCAAAATCATTC
MAPK3	RT-F	AAAGCTCCATGATGCAGCTG
	RT-R	TCAGGATTCAACGCCAAAGC
MAPK6	RT-F	GGAGAATATTCCTGCAACGTTG
	RT-R	GGAGGCTTATACTTCGCAGTAA
APX	RT-F	CGCAAAGAGGTGGAGCTAAT
	RT-R	CCTTGATAGGCTGGAGAAGTTT
CAT	RT-F	GATGAGCACACTTTGGAGCA
	RT-R	TGCCCTTCTATTGTGGTTCC
ACO1	RT-F	CATGTCCTAAGCCCGATTTG
	RT-R	TTTGAGGAGTTGAAGGCCAC
AOC3	RT-F	ACGTGCTTCTTCGGTGAAAG
	RT-R	TGGTCTTTCACTGTGGTTGC
ERF1	RT-F	TGGAGTTAGAAAGAGGCCATGG
	RT-R	CCCTCATTGATAATGCGGCTTG
EIL1	RT-F	CTGAAGATGGGCAAAGGATG
	RT-R	ACACGAGGTTGTTGATGAGG
ACS2	RT-F	AGAAAGCGCGATGAGGTTAG
	RT-R	TGGACGCAAATCCATCCAAC
ACS4	RT-F	CTGAATTCACAAATGCGATTGC
	RT-R	CAGCCATTACTACACGTTTAGC
AOC	RT-F	GAGCCAGAGCACCTCAACAGATTC
	RT-R	GCAGGGCTTCCACGATCACG
COI1	RT-F	TCTCGGAGCATCCAGCCCATATAC
	RT-R	TTGGGTCCAAAGGCTTGACAGTG
MYC2	RT-F	CAGTTTTGCCTTCTTCGGGC
	RT-R	TTCGCTGGCTTTCTACCTCG
PI-I	RT-F	GAAACTCTCATGGCACGAA
	RT-R	CCTTCGCACATCAAGTTAGAG
PI-II	RT-F	CCTATTCAAGATGTCCCCGTTC
	RT-R	GGGCAATCCAGAAGATGG
Actin	RT-F	TGGTCGGAATGGGACAGAAG
	RT-R	CTCAGTCAGGAGAACAGGGT
